# Absenteeism in healthcare settings: work-related factors and prevention

**DOI:** 10.1192/j.eurpsy.2026.11836

**Published:** 2026-07-31

**Authors:** W. Ayed, M. Mersni, S. Chemingui, Y. Yaich, G. Amri, G. Bahri, M. Bani, M. Bani, R. Ghachem, N. Ladhari

**Affiliations:** 1 Occupational department, Charles Nicolle Hospital, Tunis; 2 Psychiatric B, Razi Hospital, Mannouba, Tunisia

## Abstract

**Introduction:**

Absenteeism among healthcare workers (HCWs) is a growing concern worldwide, as it disrupts service delivery, increases workload for remaining staff, and compromises patient care quality. work-related factors play a key role in triggering or aggravating absenteeism

**Objectives:**

To describe the socio-professional characteristics of HCWs on long-term sick leave due to work-related psychiatric disorders.

**Methods:**

Retrospective descriptive study of HCWs who consulted the occupational pathology and fitness for work department, during the period from 17 August 2022 to 14 September 2025, as part of a medical examination for long-term absence.

**Results:**

Of 2404 HCWs who consulted our department for long-term sick leave, work -related factors accounted for 26,7%. The mean age of HCWs was 45,6± 10,4 years . . They were predominantly female, with a sex ratio (M/F) of 0,34. The most occupied work positions were: nurses (27,5%) and workers (16,6%). The average length of long-term sick leave was 60 days. The psychiatric pathologies most commonly reported by healthcare professionals were anxiety and depressive disorders (92%). The occupational factors reported by HCWs were high workload, shift work, workplace conflicts and inadequate organisation.

**Conclusions:**

Absenteeism among HCWs is strongly influenced by work-related factors. Our study highlight the need for targeted preventive measures and organizational interventions to reduce psychiatric morbidity and promote sustainable work capacity in healthcare settings.

**Disclosure of Interest:**

None Declared

